# Construction and validation of a prognostic model for gastrointestinal stromal tumors based on copy number alterations and clinicopathological characteristics

**DOI:** 10.3389/fonc.2022.1055174

**Published:** 2022-12-21

**Authors:** Heng Zhao, Nuohan Song, Hao Feng, Qiang Lei, Yingying Zheng, Jing Liu, Chunyan Liu, Zhengbin Chai

**Affiliations:** ^1^ Department of Oncology, Shandong Key Laboratory of Rheumatic Disease and Translational Medicine, Shandong Provincial Qianfoshan Hospital, The First Affiliated Hospital of Shandong First Medical University, Jinan, China; ^2^ Department of Research and Development, Shandong Benran Biotechnology Co., Ltd., Jinan, China; ^3^ China University of Political Science and Law, Beijing, China; ^4^ Department of Epidemiology and Preventive Medicine, School of Public Health and Preventive Medicine, Monash University, Melbourne, VIC, Australia; ^5^ Department of Clinical Laboratory Medicine, Shandong Public Health Clinical Center, Shandong University, Jinan, China

**Keywords:** gastrointestinal stromal tumors, copy number alteration, t-distributed stochastic neighbor embedding, microsatellite instability, fraction genome altered

## Abstract

**Background:**

The increasing incidence of gastrointestinal stromal tumors (GISTs) has led to the discovery of more novel prognostic markers. We aim to establish an unsupervised prognostic model for the early prediction of the prognosis of future patients with GISTs and to guide clinical treatment.

**Methods:**

We downloaded the GISTs dataset through the cBioPortal website. We extracted clinical information and pathological information, including the microsatellite instability (MSI) score, fraction genome altered (FGA) score, tumor mutational burden (TMB), and copy number alteration burden (CNAB), of patients with GISTs. For survival analysis, we used univariate Cox regression to analyze the contribution of each factor to prognosis and calculated a hazard ratio (HR) and 95% confidence interval (95% CI). For clustering groupings, we used the t-distributed stochastic neighbor embedding (t-SNE) method for data dimensionality reduction. Subsequently, the k-means method was used for clustering analysis.

**Results:**

A total of 395 individuals were included in the study. After dimensionality reduction with t-SNE, all patients were divided into two subgroups. Cluster 1 had worse OS than cluster 2 (HR=3.45, 95% CI, 2.22-5.56, *P*<0.001). The median MSI score of cluster 1 was 1.09, and the median MSI score of cluster 2 was 0.24, which were significantly different (*P*<0.001). The FGA score of cluster 1 was 0.28, which was higher than that of cluster 2 (*P*<0.001). In addition, both the TMB and CNAB of cluster 1 were higher than those of cluster 2, and the *P* values were less than 0.001.

**Conclusion:**

Based on the CNA of GISTs, patients can be divided into high-risk and low-risk groups. The high-risk group had a higher MSI score, FGA score, TMB and CNAB than the low-risk group. In addition, we established a prognostic nomogram based on the CNA and clinicopathological characteristics of patients with GISTs.

## Introduction

Gastrointestinal stromal tumors (GISTs) are the most common mesenchymal-derived tumors of the gastrointestinal tract, accounting for approximately 0.1% to 3% of all gastrointestinal malignancies, with an incidence rate of (7-15)/1 million, a prevalence age of 50-70 years, and no gender predominance (1). The vast majority of GISTs are sporadic, and approximately 5% of cases belong to familial genetic syndromes. The 5-year survival rates of restrictive, locally progressive and metastatic GISTs are approximately 93%, 80% and 55%, respectively (2). In recent years, with the continuous advancement of gene sequencing technology, the cost of sequencing has gradually decreased, which makes it possible to evaluate the prognosis of GISTs by genomic information. Compared with traditional clinicopathological information, genomic-based prognostic models are more accurate and more stable (3–5).

Copy number alterations (CNA) are the result of multiplicative amplification or deletion of DNA fragments, further affecting gene expression and thus biological phenotypes. Numerous studies have shown that CNA and tumor prognosis are correlated (6–8). Moreover, liquid biopsy technology has progressed rapidly in recent years, and circulating tumor DNA (ctDNA) has been shown to be similar to tumor tissue DNA (9). Therefore, CNA-based prognostic models may also be applied to noninvasive biopsies in the future. However, few investigators have studied the prognostic value of CNA in GISTs.

Therefore, our team used the t-distributed stochastic neighbor embedding (t-SNE) algorithm to perform a cluster analysis of the CNA dataset of patients with GISTs to establish an unsupervised prognostic model for the early prediction of the prognosis of future patients with GISTs and to guide clinical treatment.

## Method

### Study population

We downloaded the Sarcoma (MSK, 2022) dataset through a cBioPortal website (https://www.cbioportal.org/). This dataset was deeply sequenced using the MSK-IMPACT panel on 2138 sarcoma and paraneoplastic tissues. The study population was screened according to the following criteria. Inclusion criteria: (a) gastrointestinal stromal tumors confirmed by pathological diagnosis; (b) information of copy number variation was available. The exclusion criteria were as follows: (a) incomplete survival information and (b) missing clinical information.

### Variables

We extracted clinical information (e.g., age, sex), pathological information including microsatellite instability (MSI) score, fraction genome altered (FGA) score, tumor mutational burden (TMB), and copy number alteration burden (CNAB) of patients with gastrointestinal stromal tumors. For the analysis, we dichotomized continuous-type variables such as age, MSI score, FGA score, TMB, and CNAB according to the median. For the judgment of CNA, we used the GISTIC 2.0 criterion (10). This criterion uses a fixed algorithm to transform the amplification or deletion status of each gene into an integer between -2 and 2.

### Statistical method

For baseline data, if the variable was a categorical variable (e.g., sex), we used the chi-square test to detect differences in the composition ratios of different prognostic subgroups; if the variable was a continuous variable (e.g., age, MSI score, etc.), we used the Wilcoxon rank sum test to detect differences in the distribution of different prognostic subgroups. For survival analysis, we used univariate Cox regression to analyze the contribution of each factor to prognosis and calculated a hazard ratio (HR) and 95% confidence interval (95% CI). Variables with significant univariate Cox regression results were included in multivariate Cox regression for further analysis. Overall survival (OS) was defined as the time from diagnosis to the occurrence of death. For clustering groupings, we used the t-SNE method for data dimensionality reduction. Subsequently, the k-means method was used for clustering analysis. Furthermore, we drew a nomogram based on the results of multivariate Cox regression analysis, selecting factors with *P*<0.05. It was also calibrated according to 1-year OS, 2-year OS, 3-year OS and 5-year OS. All analyses were performed in R 4.1.0. GraphPad Prism 6.0 was used to generate survival curves and histograms. All statistical tests were two-sided tests. *P*-values less than 0.05 were considered statistically significant.

## Results

### Clinical characteristics in the overall population and different clusters

A total of 395 individuals were included in the study. The median age of these patients was 60 years, and the proportion of men was 54%. Notably, more than half of the patients did not have detectable CNA.

**Figure 1:**
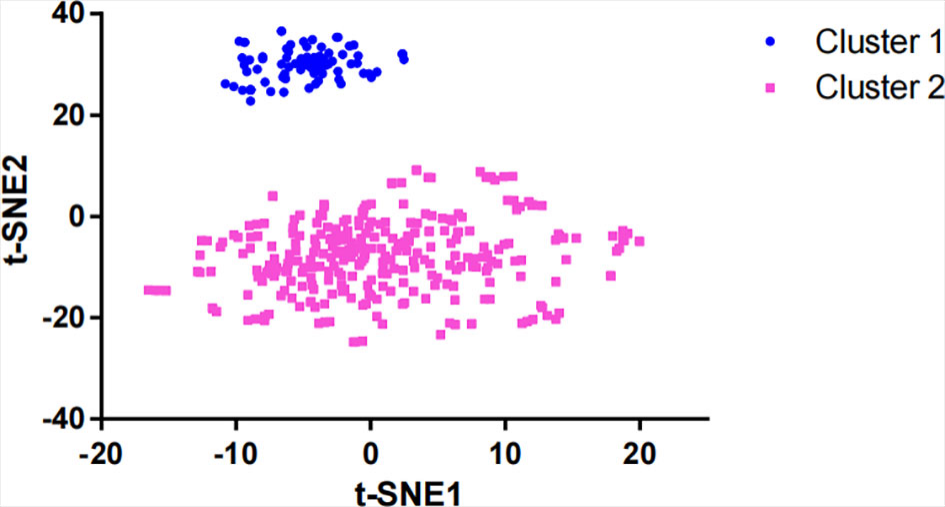
Dimension reduction of data by the t-SNE algorithm t-SNE, t-distributed stochastic neighbor embedding.

**Figure 2:**
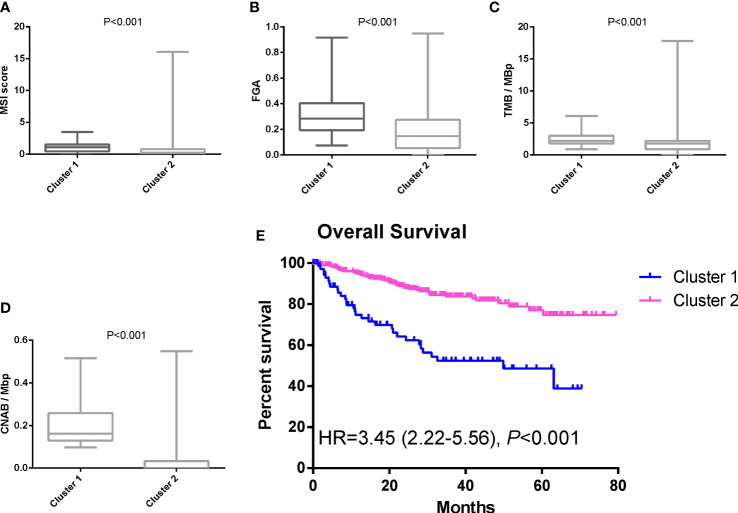
Differences in MSI score, FGA score, TMB, CNAB, and prognosis between different clusters. **(A)** MSI; **(B)** FGA; **(C)** TMB; **(D)** CNAB; **(E)** Kaplan-Meier curves. MSI, microsatellite instability; FGA, fraction genome altered; TMB, tumor mutational burden; CNAB, copy number alteration burden; HR, hazard ratio.

**Figure 3:**
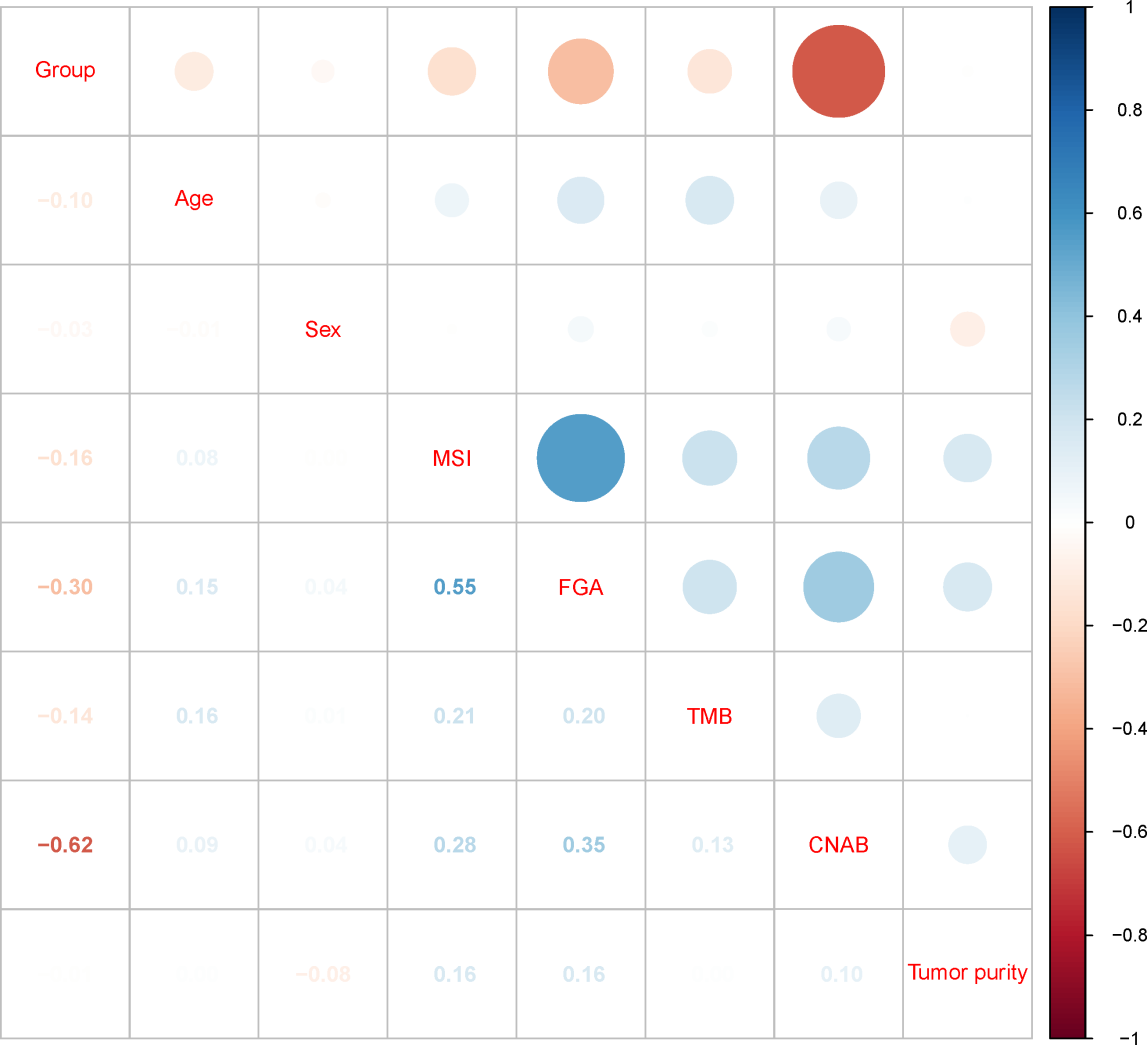
Correlation matrix of subgroups and clinicopathological characteristics. MSI, microsatellite instability; FGA, fraction genome altered; TMB, tumor mutational burden; CNAB, copy number alteration burden.

After dimensionality reduction with t-SNE (R script: set.seed=2022), all patients were divided into two subgroups (**Figure 1**). The D-index also suggested dividing all populations into 2 clusters (**Supplementary Figure 1**). The comparison of the clinicopathological characteristics of the two clusters is shown in **Table 1**. There was no statistically significant evidence confirming differences in age, sex or tumor purity between the two clusters. The median MSI score of cluster 1 was 1.09, and the median MSI score of cluster 2 was 0.24, which were significantly different (*P*<0.001, **Figure 2A**). The FGA score of cluster 1 was 0.28, which was higher than that of cluster 2 (*P*<0.001, **Figure 2B**). In addition, both the TMB and CNAB of cluster 1 were higher than those of cluster 2, and the *P* values were less than 0.001 (**Figures 2C, D**). Furthermore, we visualized the correlation coefficients between the variables (**Figure 3**). It is clear that the division of the population is highly correlated with CNAB (r=-0.62).

**Table 1:** Clinical characteristics and genomic scores.

	Overall (n=395)		Cluster 1 (n=84)		Cluster 2 (n=311)		*P*-value
	Median (Cases)	IQR (%)	Median (Cases)	IQR (%)	Median (Cases)	IQR (%)	
Sex							0.587
Female	182	46%	36	43%	146	47%	
Male	213	54%	48	57%	165	53%	
Age	60	50-68	61	54-69	59	49-68	0.103
MSI score	0.34	0.08-1.07	1.09	0.45-1.50	0.24	0.06-0.78	<0.001
FGA score	0.18	0.09-0.30	0.28	0.20-0.40	0.15	0.05-0.27	<0.001
TMB	1.8	0.90-2.60	2.2	1.80-3.00	1.8	0.90-2.20	<0.001
CNAB	0	0-0.13	0.16	0.13-0.26	0	0-0.03	<0.001
Tumor purity	70	60-80	70	60-80	70	60-80	0.962

IQR, interquartile range; MSI, microsatellite instability; FGA, fraction genome altered; TMB, tumor mutational burden; CNAB, copy number alteration burden.

### Survival analysis

**Figure 4:**
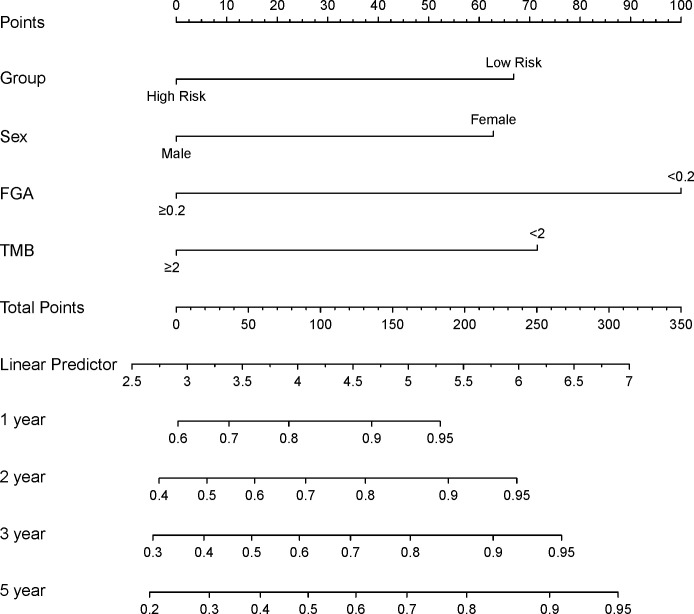
Construction of a prognostic nomogram for patients with GISTs. FGA, fraction genome altered; TMB, tumor mutational burden.

**Figure 5:**
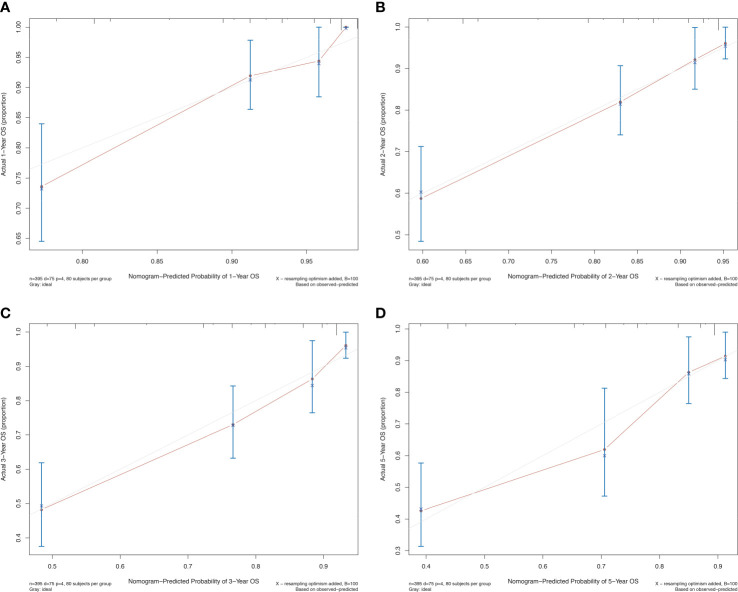
Calibration curve for the prognostic nomogram for patients with GISTs. **(A)** 1-year OS; **(B)** 2-year OS; **(C)** 3-year OS; **(D)** 5-year OS. OS, overall survival.

The prognostic differences between the two clusters are shown in **Figure 2E**. Cluster 1 had worse OS than cluster 2 (HR=3.45, 95% CI, 2.22-5.56, *P*<0.001). Therefore, we designated cluster 1 the high-risk group and cluster 2 the low-risk group. Meanwhile, we included other clinicopathological characteristics for multivariate Cox regression analysis (**Table 2**). After adjusting for sex, MSI score, FGA score, TMB and CNAB, the high-risk group still had a higher risk of death than the low-risk group (HR=1.82, 95% CI, 1.05-3.22, *P*=0.034). Furthermore, we drew a nomogram (**Figure 4**) based on the results of multivariate Cox regression analysis, selecting factors with *P*<0.05. It was also calibrated according to 1-year OS, 2-year OS, 3-year OS and 5-year OS (**Figure 5**).

**Table 2:** Survival analysis between different groups.

	Univariate Cox Regression		Multivariate Cox Regression	
	HR (95% CI)	*P*-value	HR (95% CI)	*P*-value
Group				
Low Risk	reference		reference	
High Risk	3.45 (2.22-5.56)	<0.001	1.82 (1.05-3.22)	0.034
Age				
<60	reference			
≥60	1.17 (0.74-1.84)	0.506		
Sex				
Female	reference		reference	
Male	2.01 (1.23-3.28)	0.005	1.9 (1.16-3.11)	0.011
MSI score				
Low	reference		reference	
High	3.18 (1.89-5.36)	<0.001	1.04 (0.55-2)	0.897
FGA score				
Low	reference		reference	
High	4.32 (2.56-7.27)	<0.001	2.82 (1.5-5.29)	0.001
TMB				
Low	reference		reference	
High	3.12 (1.93-5.05)	<0.001	2.33 (1.42-3.82)	<0.001
CNAB				
Low	reference		reference	
High	3 (1.83-4.9)	<0.001	1.35 (0.74-2.49)	0.331
Tumor purity				
Low	reference			
High	1.13 (0.71-1.8)	0.616		

HR, hazard ratio; 95% CI, 95% confidence interval; MSI, microsatellite instability; FGA, fraction genome altered; TMB, tumor mutational burden; CNAB, copy number alteration burden.

### Differences in CNA between different risk groups

We downloaded the CN segment plot of patients with gastrointestinal stromal tumors from the cBioPortal website (**Supplementary Figure 2**). Furthermore, the CNA status of patients with different prognostic groups was plotted as a heatmap (**Supplementary Figure 3**). The CDKN2 gene may be the relatively important gene affecting these two prognostic groups.

## Discussion

With the above analysis, we divided all patients with GISTs into two categories and distinguished prognostic grading. The high-risk group had a higher MSI score, TGA score, TMB, and CNAB than the low-risk group. In addition, we developed an OS prediction model, and the calibration curve showed a good fit.

Currently, the known factors associated with prognosis are tumor size, site, nuclear split phase count, tumor rupture, positive cut margins, KIT 11 exon deletion mutations, and other adverse biological behaviors (11–15). The Fletcher classification criteria and the Miettinen-Lasotar classification criteria (also known as AFIP risk assessment) have been used clinically as risk assessment for GISTs, the former classifying four grades of very low, low, medium and high risk with only two indicators of tumor size and nuclear split phase count, the latter adding tumor primary site to tumor size and nuclear split phase count parameter indexes and introduced a comprehensive scoring method to classify them (16). As the above two grading criteria incorporate fewer indicators for evaluation, they restrict the accuracy and clinical referability of prediction results to a certain extent. Currently, the most commonly used risk assessment systems mainly include the National Institutes of Health (NIH) criteria (2008 version), the NIH 2008 modified version (also known as the Chinese consensus 2017 modified version) and the WHO prognostic grouping criteria, among which the NIH 2008 modified version is based on a combination of indicators such as tumor size, nuclear split phase count, tumor primary site, and the presence of tumor rupture and is divided into four grades: very low, low, intermediate, and high risk. The modified version of NIH 2008 is relatively simple and practical and is the most widely used in clinical practice, but the accuracy still needs to be improved, especially because there are “very high risk” patients in the high-risk group and “medium-high risk” patients in the low-risk group. The accuracy remains to be improved.

As next-generation sequencing technology continues to evolve, an increasing number of genomic-based prognostic models are being developed (17, 18). Wei’s team incorporated immune infiltration indicators and PD-L1 to build a prognostic model for patients with GISTs through the lasso-Cox model. They found that this model not only predicted the prognosis of patients with GISTs but also the efficacy of imatinib (19). Liang et al. developed a prognostic model based on Ki-67, CD44 and PTEN expression that showed excellent prediction of disease-specific survival in patients with GISTs (20). Hiroshi’s team used the proteome and transcriptome to reveal the prognostic features of patients with GISTs at a multiomics level (21).

However, the prognostic model based on transcriptome sequencing is remarkably accurate. However, with the continuous improvement of liquid biopsy technology, the cost of ctDNA testing is getting lower. ctDNA testing, as a noninvasive test, will be more easily applied in clinical practice in the future. Considering that tissue DNA and ctDNA have some correlation, it is likely that the establishment of a DNA sequencing-based model can be applied to noninvasive liquid biopsy in the future (9).

According to further study analysis, we found that the CNA of the CDKN2A gene may be the main difference between the two groups of patients. Florian’s team found that differential regulation schemes of the CDKN2A tumor suppressor pathway converging to upregulation of E2F1 as the critical link to increased cell proliferation and adverse prognosis of GISTs (22). Michael similarly confirmed that deletion of CDKN2A/B is a poor prognostic indicator for patients with KIT mutant GISTs (23). Therefore, further fundamental experiments are needed to confirm the role of CDKN2A in the development of GISTs.

There are two potential limitations of our study. First, we did not include a validation population. In future studies, our team will include patients with GISTs in China for further validation. Second, the MSK dataset did not provide recurrence/metastasis data, so we could not evaluate disease-free survival or progression-free survival.

## Conclusion

In conclusion, based on the CNA of GISTs, patients can be divided into high-risk and low-risk groups. The high-risk group had a higher MSI score, FGA score, TMB and CNAB than the low-risk group. In addition, we established a prognostic nomogram based on the CNA and clinicopathological characteristics of patients with GISTs.

## Data availability statement

The original contributions presented in the study are included in the article/**Supplementary Material**. Further inquiries can be directed to the corresponding authors.

## Ethics statement

The study was approved by the Ethics Committee of Shandong Provincial Qianfoshan Hospital (Jinan, China). Written informed consent for participation was not required for this study in accordance with the national legislation and the institutional requirements.

## Author contributions

HZ, NS, CL, and ZC designed the study; HZ, NS, HF, QL, YZ, and JL performed the research; HZ, NS, HF, and QL analyzed the data; HZ and NS wrote the paper. All authors contributed to the article and approved the submitted version.
