# Supplementary figures and images for: Construction and validation of a prognostic model for gastrointestinal stromal tumors based on copy number alterations and clinicopathological characteristics
Source: Front Oncol. 2022 Dec 21;12:1055174. doi: 10.3389/fonc.2022.1055174 (PMC9811389; doi:10.3389/fonc.2022.1055174)

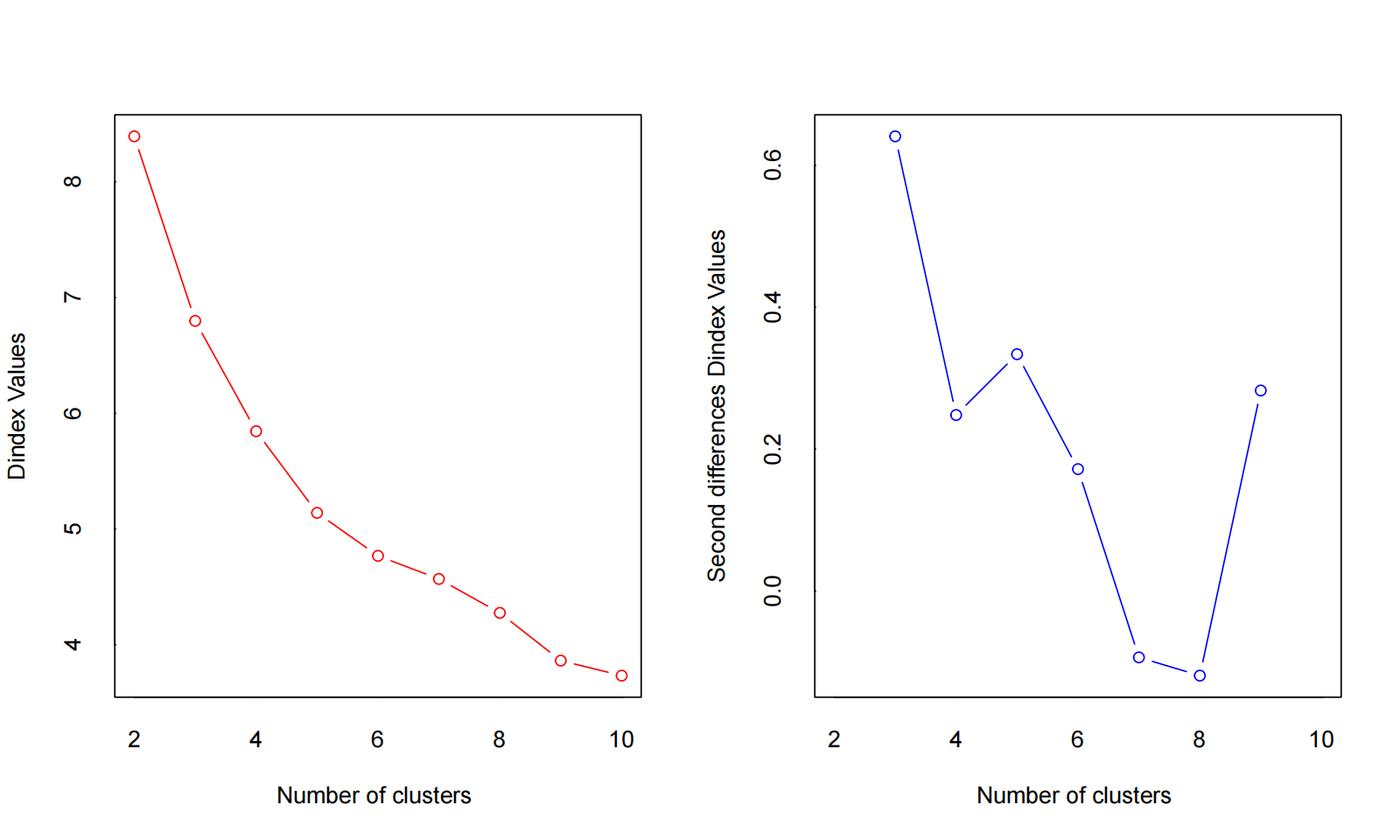

Supplement: Supplementary file 1 [file Image_1.tif]

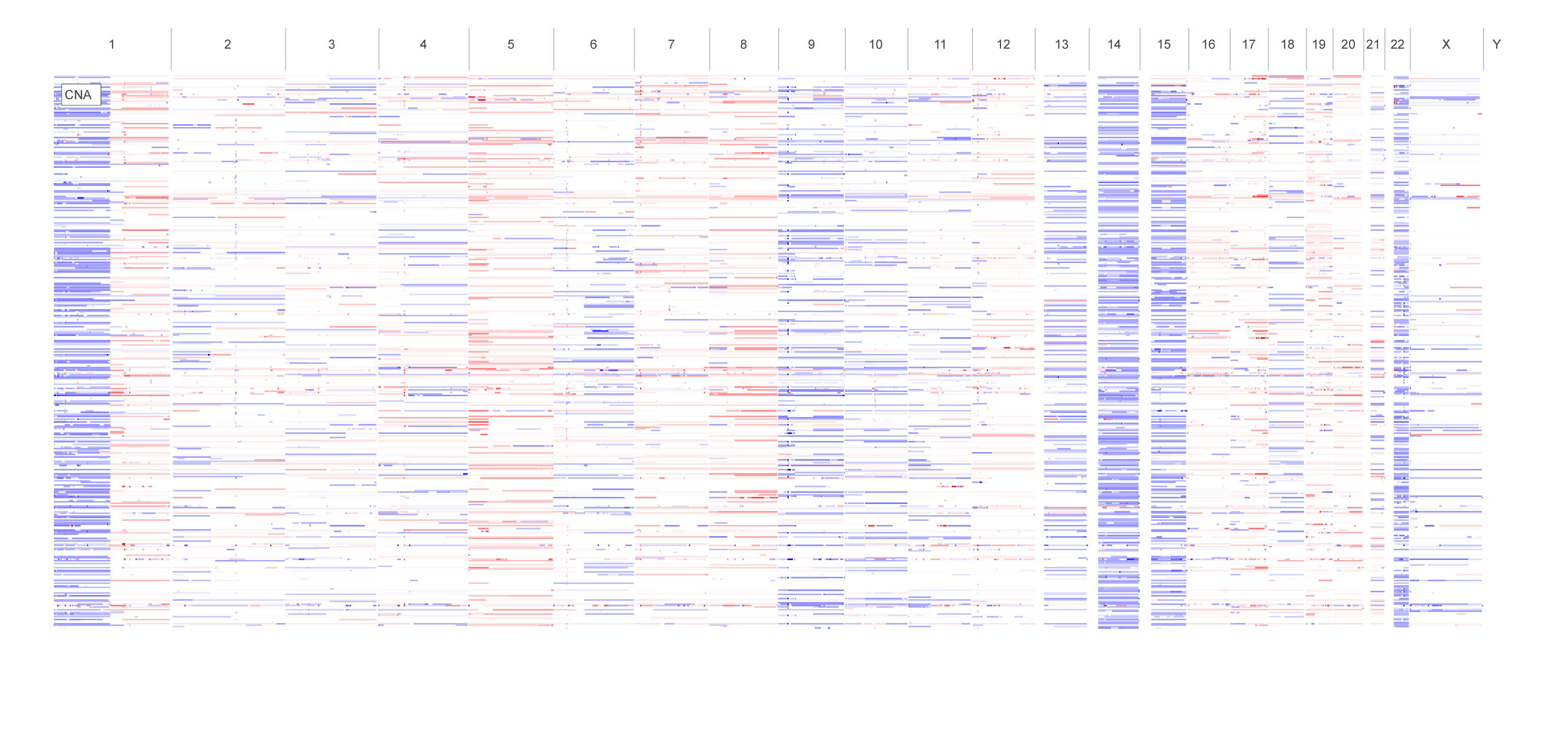

Supplement: Supplementary file 2 [file Image_2.tif]

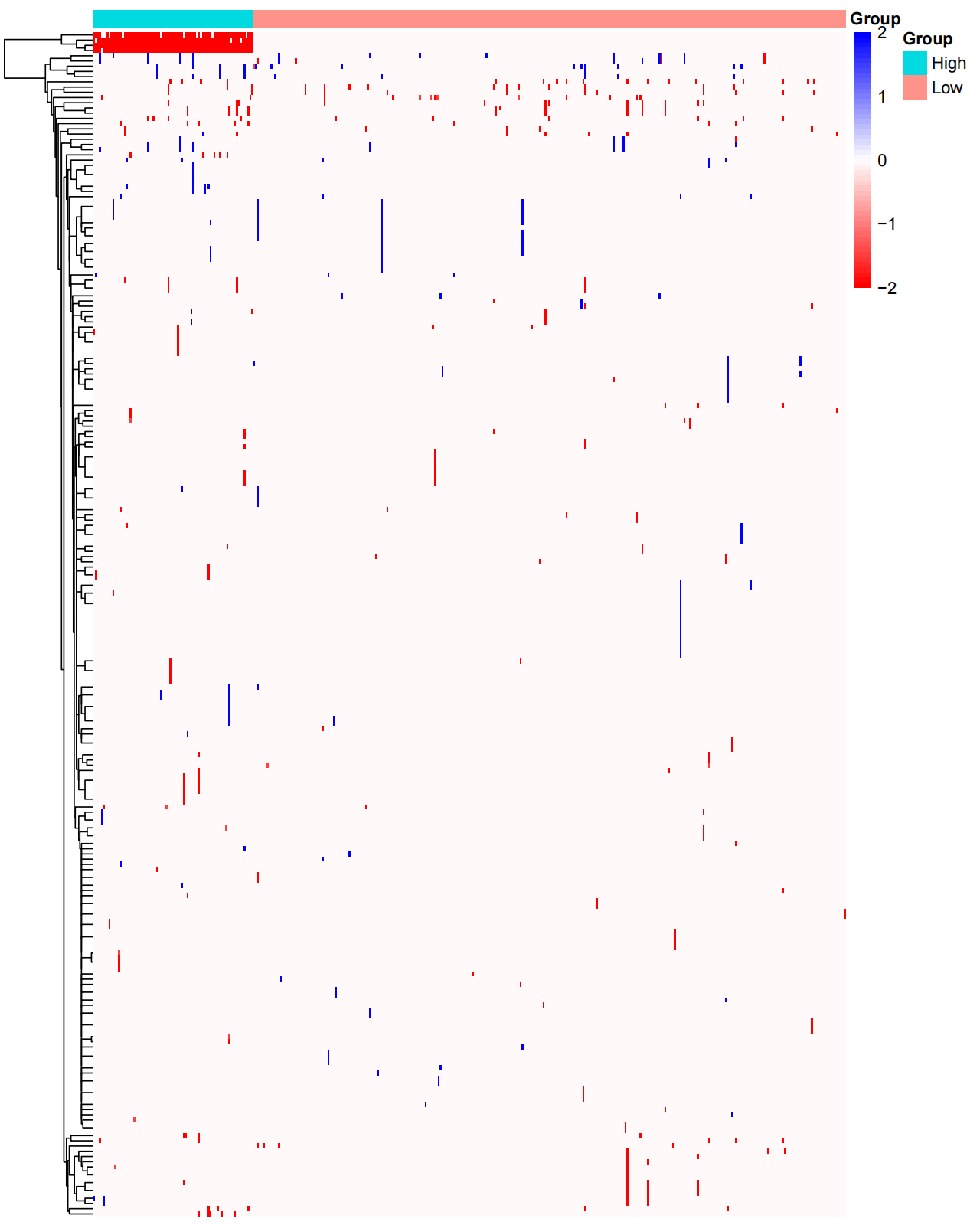

Supplement: Supplementary file 3 [file Image_3.tif]
